# Oxygen switches: Refueling for cancer radiotherapy

**DOI:** 10.3389/fonc.2022.1085432

**Published:** 2023-02-16

**Authors:** Xianghui Li, Haoran Wang, Zhiyan Li, Feng Tao, Jinhui Wu, Wenxian Guan, Song Liu

**Affiliations:** ^1^ First Affiliated Hospital of Guangxi Medical University, Depatment of Dermatology, Nanning, China; ^2^ Affiliated Nanjing Drum Tower Hospital, Nanjing University Medical School, Nanjing, China; ^3^ State Key Laboratory of Pharmaceutical Biotechnology, Medical School and School of Life Science, Nanjing University, Nanjing, China; ^4^ Department of Pharmacology, School of Pharmacy, Nanjing University of Chinese Medicine, Nanjing, China

**Keywords:** radiotherapy, tumor hypoxia, oxygen carrier, *in-situ* oxygen generation, photosynthesis

## Abstract

Radiotherapy remains the major therapeutic intervention for tumor patients. However, the hypoxic tumor microenvironment leads to treatment resistance. Recently, a burgeoning number of nano-radiosensitizers designed to increase the oxygen concentration in tumors were reported. These nano radiosensitizers served as oxygen carriers, oxygen generators, and even sustained oxygen pumps, attracting increased research interest. In this review, we focus on the novel oxygen-enrich nano radiosensitizers, which we call oxygen switches, and highlight their influence in radiotherapy through different strategies. Physical strategies-based oxygen switches carried O_2_ into the tumor *via* their high oxygen capacity. The chemical reactions to generate O_2_ *in situ* were triggered by chemical strategies-based oxygen switches. Biological strategies-based oxygen switches regulated tumor metabolism, remodeled tumor vasculature, and even introduced microorganisms-mediated photosynthesis for long-lasting hypoxia alleviating. Moreover, the challenges and perspectives of oxygen switches-mediated oxygen-enrich radiotherapy were discussed.

## 1 Introduction

Radiotherapy remains a major treatment for tumor patients (1). It is reported that 50% of tumor patients required radiotherapy (2). Oxygen serves as the fuel to stabilize DNA damage caused by radiation and prevent a DNA self-repair process (3). However, the efficacy of radiotherapy has been severely limited due to the hypoxia status in most solid tumors (4). Hypoxia not only leads to limited treatment efficacy but also causes tumor recurrence and metastasis after radiotherapy. The establishment of the hypoxia microenvironment is an outcome for multiple reasons, including tumor cell proliferation, abnormal vascular distribution, reprogrammed energy metabolism, etc. Oxygen-enriched strategies are necessary for refueling cancer radiotherapy.

To relieve hypoxia in the tumor microenvironment, various oxygen delivery strategies have been tested nowadays. Hyperbaric oxygen (HBO) inhalation cannot meet the need for radiotherapy, because the oxygen level only remained elevated for a short time (5). What is worse, combining erythropoietin treatment and radiotherapy for head and neck tumors resulted in significantly worse prognosis in patients (6). This phenomenon presumably ascribes to that squamous cell carcinoma tumor cells also express the erythropoietin receptor (7). Artificial blood including perfluorocarbon and hemoglobin-based oxygen-carrying solutions have been explored to increase the effectiveness of radiotherapy in rodent tumors (8). But contentious results for different doses and different schedules retarded clinical translation. A more precise and intelligent oxygen delivery strategy is needed.

**Figure 1:**
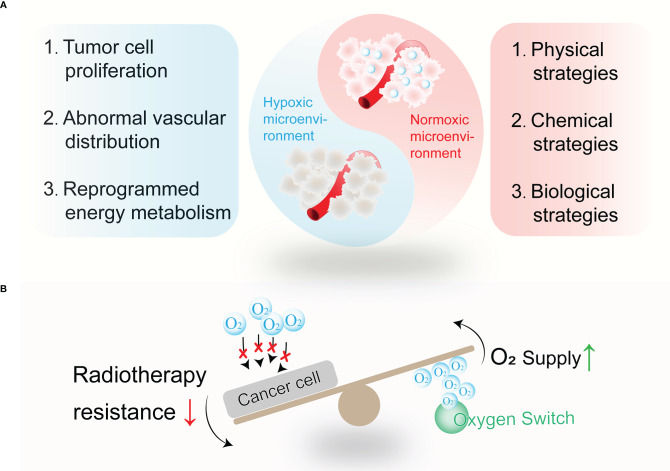
The wrestling between tumor hypoxia and related alleviating strategies. **(A)** Tumor hypoxia remains established for multiple reasons, such as tumor cell proliferation, abnormal vascular distribution, reprogrammed energy metabolism, and so on. Thus, physical strategies, chemical strategies, and biological strategies are designed to relieve tumor hypoxia. **(B)** Oxygen switches increase O_2_ supply or decrease O_2_ consumption to combat treatment resistance to radiotherapy.

To further improve the efficiency of radiotherapy, nano oxygen modulators have raised global attention. As oxygen serves as the fuel to fix radiation-induced DNA damage, radiotherapy could be boosted by evaluating oxygen concentration. In this review, a variety of agents introduced for oxygen modulation show magnifying effects for radiotherapy. Thus, we collectively refer to these nano agents as “Oxygen Switch”, which allows precise and high performance in reshaping the hypoxia microenvironment (**Figure 1**). The physical strategies including hemoglobin-based and perfluorocarbon-based oxygen carriers will be first introduced in this review, followed by a detailed discussion of chemical strategies including *in situ* H_2_O_2_ catalytic decomposition and metallic oxide decomposition. Next, we highlighted novel biological strategies such as *in-situ* photosynthesis and tumor vasculature remodeling. The mechanism and applications of these “oxygen switches” for radiotherapy enhancement are also covered. Finally, the challenges and perspectives of oxygen switches utilized in radiotherapy are presented.

## 2 Physical strategies

To relieve the hypoxia in the tumor microenvironment and improve the efficacy of radiotherapy, delivering exogenous oxygen into the tumor site as a radiosensitizer was first explored in the 1930s (9). Physical strategies refer to directly delivering exogenous oxygen *via* oxygen carriers without chemical or biological reactions. Though physical only relieves hypoxia temporally, it is enough for the radiation process *via* precise tumor targeting. To date, perfluorocarbons (PFCs) and hemoglobin (Hb) or their derivatives are regarded as excellent oxygen carriers for their high oxygen capacity and favorable biocompatibility, and emerging physical strategies-based oxygen switches are established (**Table 1**).

**Table 1:** Physical strategies-based oxygen switches.

Oxygen source	Oxygen carrier	Agent	Cancer cell types	Advantage	Ref.
Exogenous oxygen	PFC	O_2_@PFC@FHA NPs	Colon cancer	Safe and specific oxygen delivery	(10)
Exogenous oxygen	PFC	PFCE@fCaCO3-PEG	Colon cancer and breast cancer	Chemically modulating tumor hypoxic and acidic microenvironments	(11)
Exogenous oxygen	Hb	Cur@Hb	Hepatocellular carcinoma	Inhibit migration and vascular mimics	(12)
Exogenous oxygen	PFC	PFC-Q1@PLGA	Breast cancer	Synergistic whole-body therapeutic responses	(13)
Exogenous oxygen	Hb	Hb@Hf-Ce6 NPs	Breast cancer	RT-RDT in combination with immunotherapy	(14)
Exogenous oxygen	PFC	pHPFON-NO/O_2_	Glioma	On-demand temperature-controlled photothermal and oxygen-elevated radiotherapy	(15)
Exogenous oxygen	Hb	Au-Hb@PLT	Breast cancer	Combination of oxygen carrier and radiosensitizer	(16)
Exogenous oxygen	PFC	PFTBA@HSA	Colon cancer and breast cancer	Two-stage oxygen delivery	(17)
Exogenous oxygen	PFC	PFC@PLGA-RBCM	Breast cancer	Effectively deliver oxygen into tumors	(18)
Exogenous oxygen	PFC	mPEG–PLGA–PFOA	Breast cancer	Continuous supply of oxygen	(19)
Exogenous oxygen	PFC	FDC@Glo NPs	Colon cancer	The first method for FDC delivery	(20)

RT, Radiotherapy; RDT, Radiodynamic therapy.

### 2.1 PFC-based oxygen carriers

PFCs demonstrated an outstanding oxygen affinity due to their fluorine atoms in the carbon skeleton (21). Of the high biocompatibility and chemical stability, PFCs have been widely used in the clinic, such as in organ transplantation and ultrasound imaging. Once the high oxygen solubility of PFCs was found to enable tumor hypoxia alleviation, PFC-based oxygen carriers were developed to enhance radiotherapy (22). Wang et al. developed a tumor-targeted 1H, 1H-perfluorooctylamine-modified hyaluronic acid-coated perfluorocarbon oxygen carrier, O_2_@PFC@FHA (perfluorooctylamine-modified hyaluronic acid) (10). For the interaction between HA and CD44 and a large amount of oxygen dissolved in the PFC core, O_2_@PFC@FHA NPs not only improved the tumor targeting but also enabled more oxygen to reach the hypoxic area of the tumor. Moreover, the encapsulation of FHA reduced the leakage of oxygen in circulation and thereby alleviating tumor hypoxia and strengthening radiotherapy.

**Figure 2:**
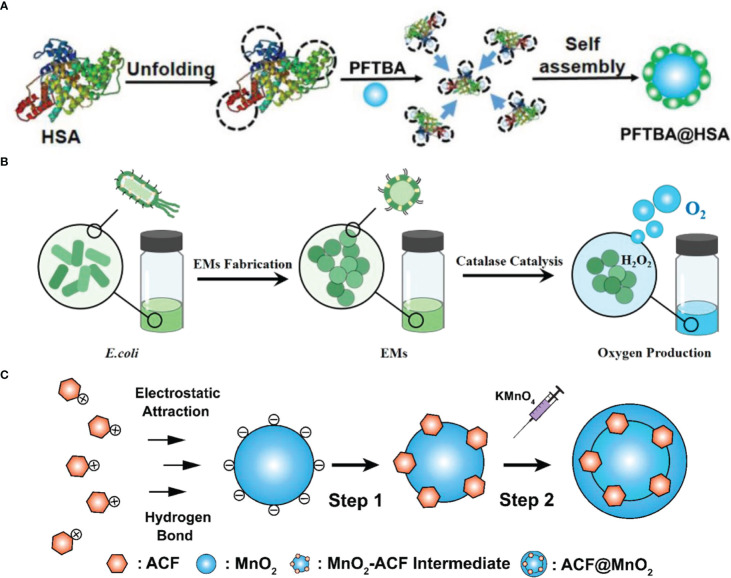
The fabrication of oxygen switches. **(A)** Schematic illustration of PFTBA@HSA preparation (17). Copyright^©^ 2021, The Author(s). **(B)** Schematic illustration of EMs preparation (3). Copyright^©^ 2021, American Chemical Society. **(C)** Schematic illustration of CAT-SAHA@PLGA preparation (23). Copyright^©^ 2021, The Author(s).

The platelet inhibition of PFC was neglected and might contribute to increasing red blood cell infiltration into tumors and improving oxygen supply. Zhou et al. screened all the perfluorocarbon compounds and found that perfluorotributylamine (PFTBA) processed the strongest platelet inhibition effect (17). Thus, the two-way O_2_ delivery system PFTBA@HSA was established, which took advantage of the platelet inhibition effect of PFTBA (**Figure 2A**). After the release of physical bound O_2_ (first step), PFTBA inhibited platelet activation and led to an increase in red blood cell (RBC) infiltration, which delivered oxygen to the tumor as the second step. This work presented a simple but effective method to reverse the resistance of tumor hypoxia to radiotherapy.

For the prolonged blood circulation time, endogenous biomimetic methods were established (24). Gao et al. developed PFC@PLGA-RBC membrane (RBCM) NPs, in which the PFC core showed efficient loading of oxygen, as well as greatly prolonged blood circulation time because of the coating of RBCM (18). The treatment efficacy during radiotherapy was remarkably enhanced for the greatly relieved tumor hypoxia. Furthermore, Yu et al. reported a nano RBC is fabricated that replaces heme with perfluorodecalin (FDC) and coated with RBCM (20). This method enabled the delivery of FDC because it cannot be emulsified by any FDA-approved emulsifiers.

### 2.2 Hemoglobin-based oxygen carriers

For the reversible and inherent oxygen-carrying capability of hemoglobin (Hb), a higher Hb level helped improved the response rate of radiotherapy (25). However, free Hb could not be directly administrated for its poor stability, which breaks the redox homeostasis and causes a severe systemic reaction. Thus, physical encapsulation or chemical conjugation is conducted to overcome these shortcomings.

Gao et al. reported that Hb and curcumin formed self-assembled nanoparticles (12). The self-assembly process was driven by hydrophobic forces and contributed to a higher cell absorption rate and lower cytotoxicity than free curcumin. Combined with the radiosensitivity of curcumin and the oxygen delivery of Hb, these nanoparticles effectively enhanced the radiotherapy for hepatocellular *in vivo*.

Hafnium (Hf), a high-Z radiosensitizer, coordinated with chlorin e6 (Ce6), and Hb was encapsulated to modulate the oxygen balance in the hypoxic TME by Wei et al. (14). The radioluminescence excited by Hf under X-ray irradiation was used to activate Ce6 for ROS generation by radiodynamic therapy (RDT). Such a multifunctional nanoplatform in the combination of oxygen supply and radiotherapy-RDT might provide a new therapeutic option for cancer eradication.

## 3 Chemical strategies

Oxygen can be generated in a large number of chemical reactions in nature. However, producing O_2_ *in vivo* by decomposing oxygenated chemicals safely and steadily is not easy. Different kinds of oxygen switches were developed, including an oxygen catalyzer or being decomposed (**Table 2**).

**Table 2:** Chemical strategies-based oxygen switches.

Oxygen source	Chemical reaction	Agent	Cancer cell types	Advantage	Ref.
Endogenous H_2_O_2_	Cu catalyzes the decomposition reaction of H_2_O_2_	RuCu NPs	Breast cancer	Combine the intrinsic nature of high-Z elements and the advantages of nanozymes	(26)
Endogenous H_2_O_2_	MnO_2_ catalyzes the decomposition reaction of H_2_O_2_	Bio-MnO_2_ NPs	NSCLC	Convert endogenic H_2_O_2_ to O_2_ and enhanced the cGAS-STING activity	(27)
Endogenous H_2_O_2_	MnO_2_ catalyzes the decomposition reaction of H_2_O_2_	PVCL-Au-MnO_2_ NGs	Pancreatic cancer	“Full-process” sensitized tumor Radiotherapy	(28)
Endogenous H_2_O_2_	MnO_2_ catalyzes the decomposition reaction of H_2_O_2_	UCNPs/CuS MnO_2_	Hepatocellular carcinoma and colon cancer	Destroy the reinforce the therapeutic effects of radiotherapy	(29)
Endogenous H_2_O_2_	Decomposition reaction of H_2_O_2_	Ce6@Leu	Hepatocellular carcinoma	LAP/GSH-driven disassembly and size shrinkage	(30)
Endogenous H_2_O_2_	Decomposition reaction of H_2_O_2_	PB reservoir and release controller	Breast cancer	Combination thermoradiotherapy	(31)
Endogenous H_2_O_2_	Pt catalyzes the decomposition reaction of H_2_O_2_	PtCo nanosphere	Lung cancer	The hollow structure amplifies the catalytic reaction	(32)
Endogenous H_2_O_2_	MnO_2_ catalyzes the decomposition reaction of H_2_O_2_	Mn-Doped Ag_2_Se nanozymes	Breast cancer	Precise radiotherapy that continuously produces oxygen	(33)
Endogenous H_2_O_2_	MnO_2_ catalyzes the decomposition reaction of H_2_O_2_	MnFe_2_O_4_-PEG	Breast cancer	Relieve hypoxia and reduce GSH concentration	(34)
Endogenous H_2_O_2_	Pt catalyzes the decomposition reaction of H_2_O_2_	BiPt-PFA	Breast cancer	Combination of photothermal therapy and enhanced radiotherapy	(35)
Endogenous H_2_O_2_	MnO_2_ catalyzes the decomposition reaction of H_2_O_2_	HSA-MnO_2_-CQ NPs	Bladder cancer	Enhanced autophagy inhibition and radiation sensitization	(36)
Endogenous H_2_O_2_	MnO_2_ catalyzes the decomposition reaction of H_2_O_2_	Cancer cell vesicle-coated MnO_2_ nanoparticles	Breast cancer	Induce cell cycle arrest in the S-phase and increases the radio-sensitivity	(37)
CuO	Decomposition reaction of CuO	IQuCs@Zr-PEG NSPs	Lung cancer	Increase the reoxygenation capacity of tumor cells	(38)
Endogenous H_2_O_2_	CeO_2_ catalyzes the decomposition reaction of H_2_O_2_	CuS@CeO_2_	Hepatocellular carcinoma	Combination of self-supplied oxygen, photothermal ability, and RT sensitive	(39)
Endogenous H_2_O_2_	Pd catalyzes the decomposition reaction of H_2_O_2_	Two-dimensional Pd@Au	Breast cancer	Sustainable and robust production of O_2_	(40)
Endogenous H_2_O_2_	MnO_2_ catalyzes the decomposition reaction of H_2_O_2_	MPDA-WS_2_ MnO_2_	Breast cancer	oxygen self-supplementing	(41)
Endogenous H_2_O_2_	Pt catalyzes the decomposition reaction of H_2_O_2_	Porous platinum nanoparticles	Large cell lung cancer	Combined advantages of a high-Z element and oxygen generation capability	(42)
Endogenous H_2_O_2_	Cu catalyzes the decomposition reaction of H_2_O_2_	Cu_2_(OH)PO_4_ nanocrystals	Cervical carcinoma	X-ray-triggered Fenton-like reaction	(43)
Endogenous H_2_O_2_	MnO_2_ catalyzes the decomposition reaction of H_2_O_2_	ACF@MnO_2_	Breast cancer	Tumor oxygenation and HIF-1 functional inhibition	(23)
Endogenous H_2_O_2_	Catalase catalyzes the decomposition reaction of H_2_O_2_	ACF-CAT@Lipo	Esophageal cancer	Oxygen enrichment and HIF-1 inhibition	(44)
Endogenous H_2_O_2_	Pt catalyzes the decomposition reaction of H_2_O_2_	AVPt@HP@M	Colon cancer	Relieving hypoxia, enhancing tumor apoptosis, and X-ray-induced photodynamic therapy	(45)
Endogenous H_2_O_2_	Catalase catalyzes the decomposition reaction of H_2_O_2_	Catalase containing E. coli membrane vesicles	Colon cancer	Catalase protection and immune stimulation	(3)
Endogenous H_2_O_2_	MOF nanohybrid catalyzes the decomposition reaction of H_2_O_2_	MOF-Au-PEG	Glioma	Enhance the radiotherapy effect and alleviate tumor hypoxia	(46)
Endogenous H_2_O_2_	Catalase catalyzes the decomposition reaction of H_2_O_2_	^131^I-Cat/CpG/ALG hybrid gel	Breast cancer	Biocompatible components enable local tumor treatments and systemic therapeutic responses	(47)
Exogenous H_2_O_2_	Catalase catalyzes the decomposition reaction of H_2_O_2_	CAT@liposome and H_2_O_2_@liposome	Breast cancer	Delivering catalase and exogenous H_2_O_2_ into tumors	(48)
Endogenous H_2_O_2_	Pt catalyzes the decomposition reaction of H_2_O_2_	Pt_2_Au_4_ cluster	Cervical carcinoma	Sustainable production of O_2_ by cluster alloying	(49)
Endogenous H_2_O_2_	CeO_2_ catalyzes the decomposition reaction of H_2_O_2_	GDY–CeO_2_ nanocomposites	Esophageal cancer	Multisensitized radiotherapy strategy	(50)
Endogenous H_2_O_2_	Catalase catalyzes the decomposition reaction of H_2_O_2_	PLGA-R837@Cat nanoparticles	Breast cancer and colon cancer	Synergistic whole-body therapeutic responses after local treatment	(51)
Endogenous H_2_O_2_	Catalase catalyzes the decomposition reaction of H_2_O_2_	CAT-SAHA@PLGA	Colon cancer	Synergistically increasing tumor oxygenation and inhibiting HDAC activity	(52)
Endogenous H_2_O_2_	Carbon substrate catalyzes the decomposition reaction of H_2_O_2_	Hf-MOF	Breast cancer	peroxidase-like activity and distinct NIR-II absorption properties	(53)

CAT, catalase; A, Apoptin; V, verteporfin; HP, Hollow polydopamine; M, cancer cell membrane; MOF, Metal-organic framework; GDY, 2D graphdiyne.

### 3.1 Oxide-based oxygen generator

Early leakage of delivering exogenous O_2_ into the tumor site remains a challenge for physical strategies. Thus, delivering the precursors of oxygen to the hypoxic area and generating O_2_ *in situ* is attractive.

Chen et al. utilized CuO nanoparticles to generate O_2_ under microwave irradiation in the tumor microenvironment (38). Decorated with MW sensitizer 1-butyl-3-methylimidazolium hexafluorophosphate (IL) and radiosensitizer of Quercetin (Qu), the mesoporous sandwich SiO_2_@ZrO_2_ nanoparticles (SiO_2_@ZrO_2_ NPs) persistently released oxygen under MW irradiation, which significantly increased the re-oxygenation ability of tumor cells. Due to the reshaping of the tumor microenvironment, a high inhibition rate of 98.62% was witnessed in the *in vivo* anti-tumor experiment.

### 3.2 Catalyzer-based oxygen generator

Due to the abnormal metabolism and redox homeostasis, a high level of H_2_O_2_ is found in the tumor cells compared to normal cells. To generate O_2_ *via* H_2_O_2_ decomposition, catalase (CAT) and nano-enzyme-loaded oxygen switches were developed to enhance radiotherapy *in situ*.

CAT generates oxygen *in situ* but may be degraded *in vivo* due to the upregulated protease in the tumor. Zai et al. developed highly protease-resistant *E. coli* membrane vesicles (EMs) to contain CAT and thus relieve tumor hypoxia for a long time (3) (**Figure 2B**). EMs demonstrated a higher CAT activity than free CAT even in the concentration of 100-fold protease. Combined with immune stimulation features, EMs maintained their hypoxia relief ability for a long time and enhanced radiotherapy.

Song et al. developed a strategy that delivers exogenous H_2_O_2_ to the tumor microenvironment and subsequent CAT-triggered H_2_O_2_ decomposition (48). CAT and H_2_O_2_ were separately encapsulated within stealthy liposomes for a long-lasting effect in tumor re-oxygenation enhancement. Furthermore, the relieved tumor hypoxia enhanced the therapeutic effects of radiotherapy and reversed the immunosuppressive tumor microenvironment. Combined with CTLA4 blockade, the radio-immunotherapy induced effective anti-tumor immune responses to destruct tumors.

Zhang et al. reported a nanoplatform based on poly(N-vinyl caprolactam) (PVCL) nanogels (NGs) co-loaded with gold (Au) and manganese dioxide (MnO_2_) nanoparticles (NPs) for sensitized radiotherapy (28). MnO_2_ displayed the CAT-mimic catalytic activity that decomposed H_2_O_2_ to form O_2_ and alleviate tumor hypoxia (**Figure 2C**). Resulted Mn^2+^ exerted a Fenton-like reaction to cause intracellular ROS and made the cells more susceptible to radiotherapy. Meanwhile, Au NPs and Mn (II) transformed from MnO_2_ NPs guided the *in vivo* radiotherapy through dual mode CT/MR imaging.

## 4 Biological strategies

Though chemical strategies display a prolonged oxygen modulation capacity than physical strategies, the hypoxic environment reappears once the chemicals are exhausted. Since tumor hypoxia is the outcome of abnormal biological behavior of tumor cells, emerging biological strategies-based oxygen switches may provide exciting opportunities and should be highlighted (**Table 3**).

**Table 3:** Biological strategies-based oxygen switches.

Oxygen source	Biological behavior	Agent	Cancer cell types	Advantage	Ref.
Reduced oxygen consumption	Inhibit mitochondria respiration	Hf-PSP-DTC@PLX	Breast cancer	Synergistic strategy for improvement of oxygenation and oxygen utilization	(54)
Reduced oxygen consumption	Inhibit mitochondria respiration	AuNCs- PEG-SNP-PM	Colon Cancer	NO inhibited cell respiration and O2 consumption	(55)
Increased blood perfusion	Remold tumor vasculature	AuHQ nanoparticles	Hepatocellular carcinoma	Alleviating tumor hypoxia and increased blood perfusion	(56)
Increased blood perfusion	Remold tumor vasculature	NO depot	Melanoma	provide low dosage NO continuously and release a large amount of NO immediately before irradiation for a short time	(57)
Photosynthesis	Microalgae-mediated photosynthesis	RBCM-Algae	Breast cancer	*In situ*–generated oxygen and ROS	(58)
Photosynthesis	Cyanobacteria-mediated photosynthesis	Cyanobacteria-loaded bismuthene nanosheets	Breast cancer and Lung cancer	Photosynthetic hypoxia-alleviation capability and radiosensitization performance	(59)
Photosynthesis	Cyanobacteria-mediated photosynthesis	Photosynthetic microcapsules	Melanoma	Evoked lipid peroxidation, Fe^2+^ release, GPX4 suppression, glutathione reduction, and ferroptosis	(60)
Photosynthesis	Microalgae-mediated photosynthesis	Algae@SiO_2_	Breast cancer	PAI/FI dual imaging, radiosensitization, and cascaded photothermal therapy	(61)
Reduced oxygen consumption	Inhibit mitochondria respiration	“Nano-boat”	Breast cancer	Efficiently induce cancer cell apoptosis by the energy metabolism inhibition involving multiple pathways	(62)

GPX4, glutathione peroxidase 4.

### 4.1 Oxygen switches to decrease oxygen consumption

To support accelerated cell proliferation, excessive oxygen consumed is one of the critical reasons for tumor hypoxia. Thus, metabolism regulation-based strategies that inhibit tumor aerobic respiration is promising.

As mitochondria refer to the energy house that consumes oxygen to generate energy for tumor growth, mitochondria-targeted interventions are believed to enhance radiotherapy. Gao et al. fabricated a mitochondria-targeted nano-platform *via* the integration of a self-assembled peptide and a positively charged cyclen (62). The positively charged cyclen anchored to the mitochondria and loaded lonidamine, which served as the energy stripper of cancer cells, inhibited energy metabolism and oxygen consumption. Combined with radiotherapy and endogenous apoptosis pathway, this mitochondria-targeted intervention led to tumor eradication *in vivo*.

Recently, therapeutic gases were attractive and found to exhibit regulation effects. Nitric oxide (NO), hydrogen sulfide (H_2_S), and carbon monoxide (CO) were utilized in tumor treatment for their therapeutic capacity. Duo et al. designed an irradiation-triggered NO-release nano-prodrug to improve radiosensitization (62). Through the reaction of sodium nitroprusside and L-glutathione, high content of NO was released and thus inhibited cell respiration and oxygen consumption. Then O_2_ accumulation improved the therapeutic outcomes under irradiation by generating more ROS in the tumor microenvironment. Besides, H_2_S was also employed as an oxygen switch to remodel oxygen metabolism by inhibiting cytochrome c oxidase activity in a high-Z metal ion-sensitized radiotherapy (54).

### 4.2 Oxygen switches to increase oxygen supply

One important reason for tumor hypoxia is the abnormal blood vessel. To obtain nutrients for growth and to metastasize, tumor blood vessels are leaky, tortuous, and saccular (63). Thus, Tumor vascular normalization is a promising method to increase blood perfusion and relieve tumor hypoxia. Wang et al. modified Au NPs with 8-hydroxyquinoline (HQ) to obtain AuHQ, which attenuated the expression of angiopoietin-2, vascular endothelial growth factor 2 (56). Moreover, AuHQ treatment increased pericyte coverage and modulated tumor leakage, which led to increased blood perfusion. Tumor vascular normalization not only alleviated tumor hypoxia but also contributed to an increased AuHQ accumulation. Ultimately, compared to Au NPs, the anti-tumor efficacy of radiotherapy was increased by 38% in the AuHQ group.

Apart from chemical strategies, generating O_2_ *in situ* could be achieved by biological strategies *via* a natural photosynthetic system (64, 65). Qiao et al. engineered RBCM to modify the algal surface and deliver this RBCM-Algae to the tumor to increase tumor oxygenation (58). With red light-induced photosynthesis, RBCM-Algae generated O_2_ *in situ* and alleviated tumor hypoxia, and further led to re-oxygenated radiotherapy. Cyanobacteria were also utilized for continuous photosynthetic oxygen evolution in a two-dimensional bismuthene platform with high-Z components, which demonstrated the photosynthetic hypoxia-alleviation capability and radiosensitization performance (59). These works exemplified the construction of microorganism-enabled oxygen switches for radiosensitizer-augmented radiotherapy.

## 5 Challenges and perspective

Given the central role of tumor hypoxia in the treatment resistance to radiotherapy, a various of oxygen switches were fabricated based on physical strategies, chemical strategies, and biological strategies. In this review, we summarized the oxygen switches designed for hypoxic-tumor radiotherapy. Physical oxygen switches served as a high-capacity carrier to deliver exogenous O_2_. Chemical oxygen switches triggered an *in-situ* reaction to generate O_2_ *in vivo*. Biological oxygen switches reshaped the tumor microenvironment by regulating biological behavior or introducing microorganism-mediated photosynthesis. Over the past few years, prosperous designs gained much attention and gratifying results in pre-clinical experiments, but there remained several challenges to be addressed.

The first limitation refers to the efficacy of these oxygen switches. As known, the hypoxic tumor area is quite complex and exhibits steric heterogeneity. On the one hand, deepen understanding of the mechanism of tumor hypoxia and radiosensitization are necessary. On the other hand, oxygen switches have to improve the O_2_ concentration continuously and accurately. Bio-mimic encapsulation was employed in oxygen switches to avoid early leakage and immune clearance (20). Stimulus-response (near infrared-triggered, irradiation-triggered, focused ultrasound-triggered, etc.) oxygen switch helped to achieve spatiotemporal specificity oxygen generation (62). As the penetration depth of NIR was limited, irradiation-triggered or focused ultrasound-triggered oxygen switch was worth further exploration (66). Moreover, novel *in-situ* microorganism-mediated photosynthesis could generate O_2_ continuously. However, it is still a challenge to prolong the survival time of microorganisms.

Secondly, the biosafety and biocompatibility of these oxygen switches are of concern. Unexpected Hb exposure can cause severe side effects including blood clot formation, renal toxicity, and cardiovascular complications (67). The metal oxide may affect intracellular redox homeostasis and induce macromolecule dysfunction. Especially, CAT-mimic nano-enzyme enables activating the matrix metalloproteinases in the tumor tissue, which could lead to inflammation and even tumor metastasis (68). Introducing microorganism into a human may activate the unfavorable immune response and induces local microbiome disturbances (69).

Finally, the coordination of oxygen switches and radiotherapy should be strengthened. The pharmacokinetics of oxygen switches should be tailored to the conduction of radiotherapy. Since radiotherapy is an oxygen-consumed intervention, the amount of oxygen generated from the oxygen switches has to be precisely measured to keep the balance of oxygen concentration and reach the best outcome. Furthermore, the combination of other therapeutic interventions such as photodynamic therapy and RDT may improve the utilization efficiency of oxygen.

Researchers paid much effort to the approach of oxygen-enriched radiotherapy. Nowadays, novel technologies are paving the path to more precise clinical medicine. Single-cell sequencing helped us understand how hypoxia is shaped and the evolutionary landscapes of tumor genomics (70). Nano-robots can follow a specified route and directionality under control, thus leading to a more precise oxygen delivery (71). Genome editing may improve the safety and oxygen-generating capability of microorganisms-mediated photosynthesis (72).

To be concluded, from the first attempt to combat hypoxia for radiotherapy to enhancement, multiple strategies have been developed to increase available oxygen. Though different strategies work on different principles, the efficacy of these oxygen switches has been widely recognized. However, there remain some obstacles before clinical translation. With a more in-depth understanding of tumor hypoxia, we should believe that better radiosensitized oxygen switches will emerge.

## Author contributions

XL, HW, ZL, and FT wrote the manuscript. SL, JW, and WG revised the manuscript. All authors contributed to the article and approved the submitted version.
